# Do we need psychiatric asylums again? - Con speaker

**DOI:** 10.1192/j.eurpsy.2025.83

**Published:** 2025-08-26

**Authors:** R. Keet

**Affiliations:** GGZ Noord-Holland-Noord, Overveen, Netherlands

## Abstract

**Abstract:**

The expansion of institutional long-term psychiatric care, while well-intentioned, repeats the historical failures of the 19th century answer to severe mental illness: asylums—facilities often marred by neglect, abuse, and the marginalization of individuals with mental illness. Many of the handicaps of persons living in these asylums were not the consequence of the mental health disorder but were iatrogenic damage done by living in these asylums. Though modern psychiatric institutions are more regulated, institutionalization inherently limits autonomy and reinforces stigma, undermining recovery-oriented models of care. Instead of diverting resources to rebuild large-scale facilities, investment should focus on strengthening community-based services, supportive housing, individual placement and support and integrated care models that empower individuals with serious mental illness to live fulfilling lives within society. Evidencebased outpatient programs, Flexible Assertive Community Treatment teams, peer support networks, recovery colleges, mobile crisis teams, peer farms, respite houses offer more humane, cost-effective, and person-centered alternatives. Addressing systemic challenges—such as poverty, lack of housing, lack of social connectedness and fragmented healthcare systems—can better serve this population without reverting to institutional confinement. Long-term solutions must prioritize dignity, autonomy, and inclusion rather than a return to segregated care.

**Disclosure of Interest:**

None Declared

